# Surgical Pitfalls in Bertolotti’s syndrome management: A case report

**DOI:** 10.1097/MD.0000000000032293

**Published:** 2022-12-16

**Authors:** Hatem Afana, Muhammad Raffat, Nicandro Figueiredo

**Affiliations:** a Orthopaedic Surgeon, Department of Orthopaedic & Spinal Surgery, King’s College Hospital London, Dubai, UAE; b Spinal Neurosurgeon, Department of Orthopaedic & Spinal Surgery, King’s College Hospital London, Dubai, UAE; c Medical School, Federal University of Mato Grosso (UFMT) and University of Cuiaba (UNIC), Cuiaba, MT, Brazil.

**Keywords:** Bertolotti’s syndrome, case report, lumbosacral transitional vertebrae, minimally invasive surgery, transverse processectomy, tubular spinal surgery

## Abstract

Bertolotti’s syndrome is one of the differential causes of low back pain, especially within young people. The etiopathogenesis of the typical paramedian low back pain, associated with Bertolotti’s syndrome remains controversial, and there is no worldwide acceptance of treatment.

**Patient concerns:**

This article presents the authors experience with surgical treatment of symptomatic patients with Bertolotti’s syndrome.

**Diagnoses:**

Retrospective study of a selected series of patients with symptomatic Bertolotti’s syndrome submitted to surgical treatment.

**Interventions:**

This study included 16 patients, being 8 submitted to the new modified mini-open tubular microsurgical transverse processectomy, Among those patients, intraoperative fluoroscopy was used in 6 surgeries to locate the base of the enlarged transverse process (6/8); intraoperative neuromonitoring was used in 6 patients (6/8), 3D intraoperative advanced spinal image (O-arm) with neuronavigation was used to localize the base of the pseudojoint to be removed and to check the final bone resection for the last 5 cases (5/8).

**Outcomes:**

The average paramedian lower back pain before surgery on the visual analogue scale for pain in the 8 patients was 6.6 (range: 5–8) and reduced to 1.5 (range: 0–3) at the latest follow-up after surgery, while the average pain score of the radicular pain on the right or left side before the surgery was 1.3 (range: 0–6) and reduced to 0.6 (range: 0–7) after the surgery.

**Lessons:**

The mini-open tubular microsurgical transverse processectomy seems to be potentially safe and effective for the surgical treatment of selected symptomatic patients with Bertolotti’s syndrome.

## 1. Introduction

Low back pain (LBP) is a widespread complaint, it is a leading cause of activity limitation and absenteeism from work.^[1]^ Lumbosacral transitional vertebra (LSTV) is the most common congenital spinal vertebra anomaly at the lumbosacral junctional area, it can be associated with low back pain, but the majority of LSTV cases are asymptomatic.

The coexistence of the anomalous at lumbosacral junction and LBP was first described by Mario Bertolotti, Italian neurologist and radiologist, in 1917.^[2]^

The reported prevalence of Bertolotti’s syndrome (BS) ranges from 5 to 30%, this wide range can be explained by using different image studies done for symptomatic patient with low back pain.,^[3–10]^.

LSTV and BS can be associated with other anatomical variations, like displacement of lumbosacral plexus position and subsequent change in nerve distribution,^[11–14]^ or abnormal position of large blood vessels.,^[15–17]^. Erken et al claim that the presence of a cervical rib might be a clue to the existence of sacralizatioof *L*5 or vice versa^[18]^, while Nakajima et al proposed that there is a strong association between lumbar ribs and lumbarisation^[19]^.

The etiopathogenesis of the typical paramedian LBP, associated with Bertolotti’s syndrome remains controversial, and there is no worldwide acceptance of treatment.^[20]^ This article presents the authors experience with surgical treatment of symptomatic patients with BS.

## 2. Method

Retrospective study of a selected series of patients (16 patients) with symptomatic BS submitted to surgical treatment. All included patients were initially submitted to a detailed clinical and imaging assessment (lumbosacral X-ray, CT and MRI) for the proper identification of the enlarged transverse process (TP) and the potential painful pseudoarticulation between the TP and sacrum, and to stablish the Castellvi radiographic classification system.^[2]^

Upon the diagnosis of BS, patients were submitted to conservative treatment for at least 6 months, including the use of analgesics and physical therapy, patients with chronic and significant paramedian low back pain, with or without radicular pain, and considering surgical treatment, the pseudojoint injection test was suggested. We injected only into the potential painful pseudoarticulation joint, in which the temporary improvement of the pain was considered essential for surgical indication, in which the temporary improvement of the pain was considered essential for surgical indication. All cases who underwent surgery to treat Bertolotti’s syndrome were included in this study, with or without another simultaneous lumbar surgery to treat associated disc disease, like microdiscectomy, decompression or fusion. The surgeries were performed by the same senior neurosurgeon author, during the period of 2017 until 2022.

## 3. Initial management and injection test

To confirm the diagnosis, the patients had injection into the malformed pseudojoint between the TP and sacrum, under fluoroscopy, using local anesthesia, 2 to 5 mL of lidocaine (2%), being pseudo joint localized with contrast injection, followed by injection of 10 mL of bupivacaine (0.5%) with 40 mg of Methylprednisolone in the pseudojoint. A temporary localized pain relief was considered a successful diagnostic test block. This was used as a mandatory test for indication for the surgery. As part of the team’s treatment algorithm, the injection was performed only at the pseudo-articulation, no other spinal injection was done 1 month apart from the diagnostic test block.

## 4. Mini-open tubular microsurgical transverse processectomy (Mo-tmtp) procedure

**Figure 1.**
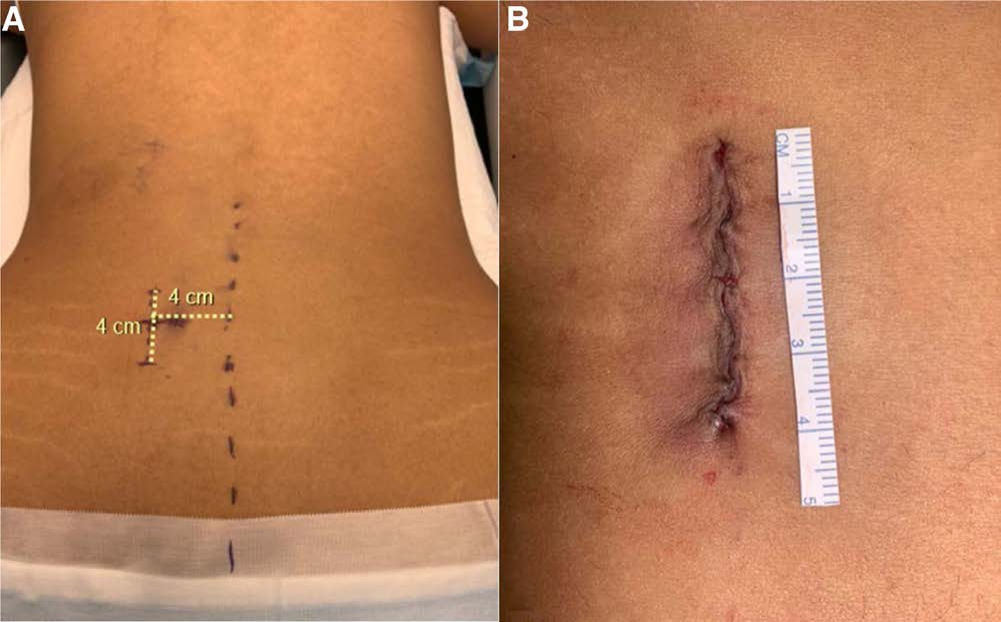
(A) Marking the skin incision, +4 cm long at the left paravertebral (vertical) region, +4 cm from the midline, confirmed by fluoroscopy; (B) Incision closed after surgery.

Patient in prone position, on the Wilson frame attached to the spinal table, under general endotracheal anesthesia, prophylactic antibiotic, intraoperative neuromonitoring, full protection of the body, face-eyes, anti DVT (deep venous thrombosis) mechanical and warming devices were also used. Using fluoroscopy, the level of base of the malformed transverse process pseudo articulation was marked approximately 4 cm long paravertebral incision, ipsilateral to the malformed joint, around 4 cm from the midline (Fig. 1). Standard antiseptic preparations, sterilization, drapes were used in the usual sterile fashion.

Real-time 3D intraoperative images system combined with the neuronavigation was used in the most recent samples of the series (since August 2020), fixing the reference frame to the lumbar spinous process (*L*4, *L*5 or LSTV), posterior superior iliac spine (PSIS), or taped-fixed to the skin. The 3D intraoperative images with neuronavigation were useful to localize the base of the TP to be respected, and to check later if the bone resection was enough to completely disconnect the malformed TP from the potentially painful pseudojoint, keeping a bone cleft or a gap of at least 5 to 10 mm between the medial part of the TP (close to the pedicle) and the lateral disconnected part of the enlarged TP-pseudojoint, to avoid late bone reunion.

**Figure 2.**
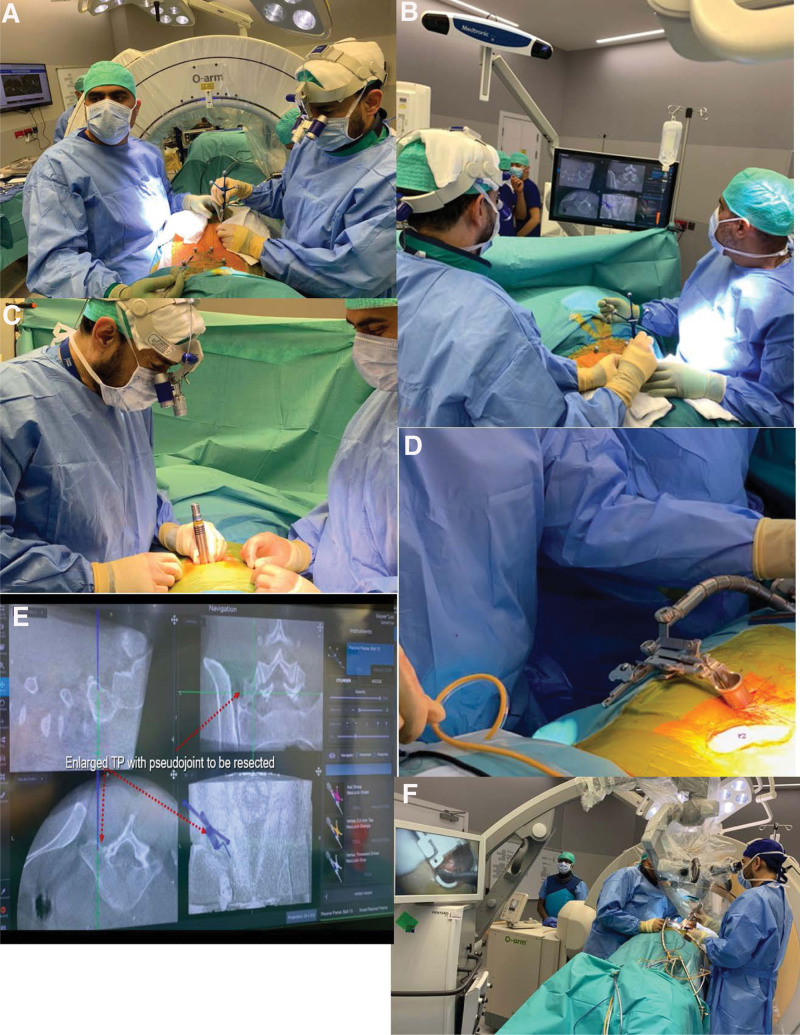
(A, B) Marking the incision using intraoperative image guidance (*O-arm*, Medtronic^®^, USA), neuronavigation with the reference frame taped on the skin (*S8*, Medtronic^®^, USA) and checking the location on the screen; (C) Percutaneous paravertebral surgical access using series of sequential muscle dilators at the base of the transverse process (TP); (D) Tubular retractors docking on the inferior facet-joint on the top of the L5 enlarged TP for the transverse processectomy and fixed to the spinal table; (E) Neuronavigation probe guiding and confirming the location of the enlarged TP to be partially resected; (F) Tubular- transverse processectomy being performed using microsurgical technique, guided by 3D image (*O-arm*, Medtronic^®^), neuronavigation (*S8*, Medtronic^®^) and intraoperative neuromonitoring (IONM, *NIM-eclipse*, Medtronic^®^, USA). IONM = intraoperative neuromonitoring, TP = transverse process.

A vertical paravertebral incision (*+*4 cm) was performed ipsilateral to the malformed joint, directly over the region of the pseudoarticulation, *+*4 cm from the midline (Fig. 1). The fascia was opened sharply, followed by placement of a series of sequential muscle dilation guides, cannulas, and tubes (*MAST-quadrant system*^*™*^, Medtronic^®^, Minneapolis). Tubular retractors aiming to the involved TP were assembled, which confirmed that the retractors were docked on the base of the malformed TP by fluoroscopy or *O-arm* with neuronavigation, and then the retractors were fixed to the operating room table (Fig. 2).

The soft tissue was cleaned on the surface of the enlarged TP, exposing the base of the malformed TP to be partially resected. At this stage, the surgical loupe was replaced by a surgical microscope for the proper bone removal and disconnection of the enlarged TP-pseudojoint from the lumbar segment of the spine. It was used high speed drill (*Midas Rex*^*™*^, Medtronic^®^, Minneapolis) for the superficial part of the bone removal, and ultrasound bone scalpel (*Misonix*^*®*^, Farmingdale) for the deeper part, to lessen the risk of nerve or vascular injury.

To facilitate the partial TP resection to disconnect it from the remaining malformation, in special to avoid any pressure to the painful pseudojoint, the modified technique that this article proposes has the following key points: start resecting the enlarged TP vertically, lateral to the base of the TP, superior-inferior; creating a bone gap of at least 5 to 10 mm; after passing the level of the pedicle, the bone resection is redirected inferior-medially, below the pedicle, a “ice hockey stick” shape, around 1 cm from the base of the TP, keeping the pedicle intact medially and the enlarged TP-pseudojoint laterally.

**Figure 3.**
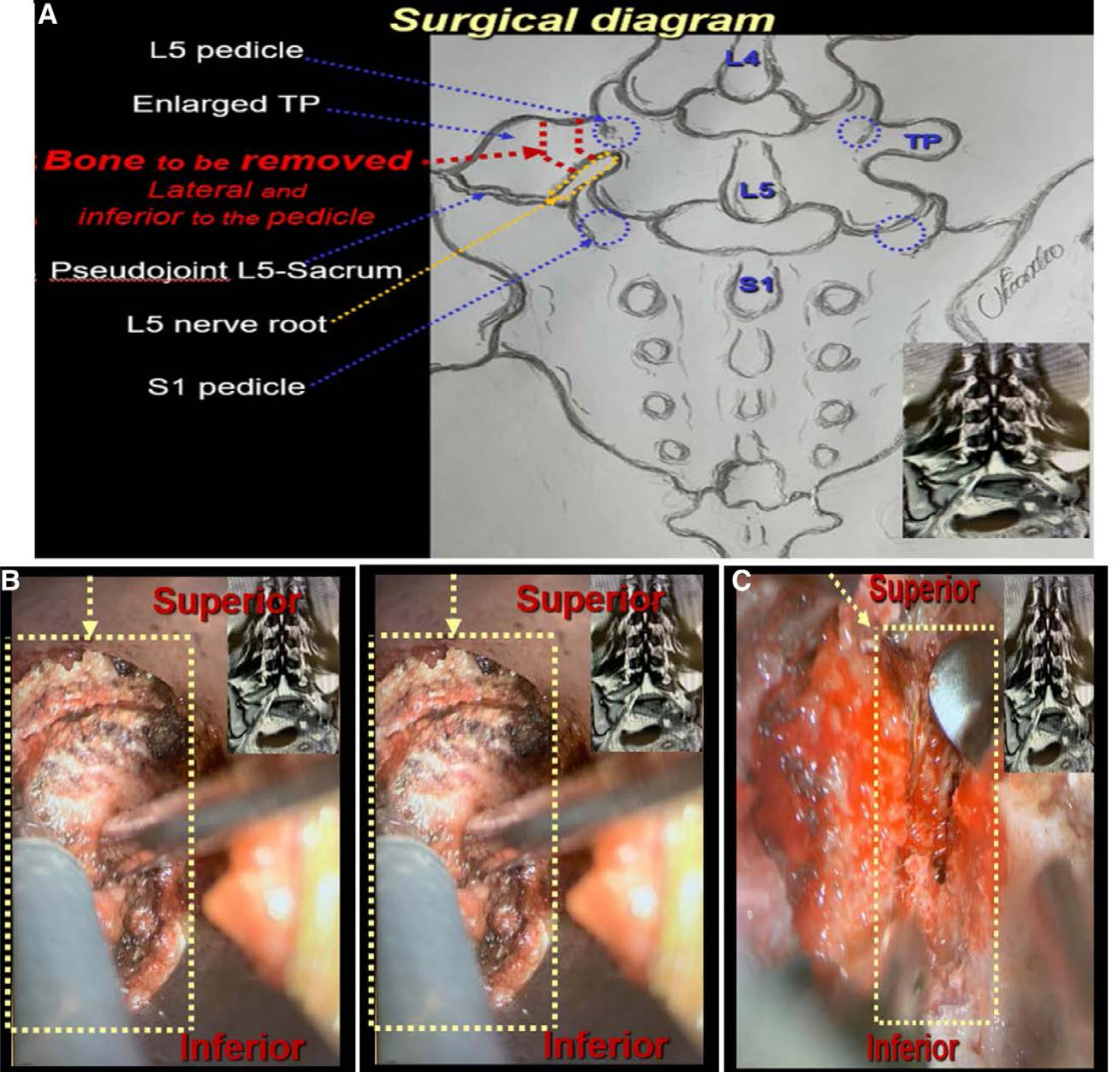
(A) Diagram demonstrating the modified transverse processectomy: vertical bone resection creating a gap (>5 mm) at the base of the enlarged transverse process (TP), lateral and parallel to the pedicle, followed by an oblique and inferior bone resection, separating the malformed TP from the pedicle of the lumbar vertebra; (B) Microscopic intraoperative view exposing the area of the resection; (C) During the transverse processectomy; (D) and After the bone resection for disconnection of the enlarged TP. TP = transverse process.

**Figure 4.**
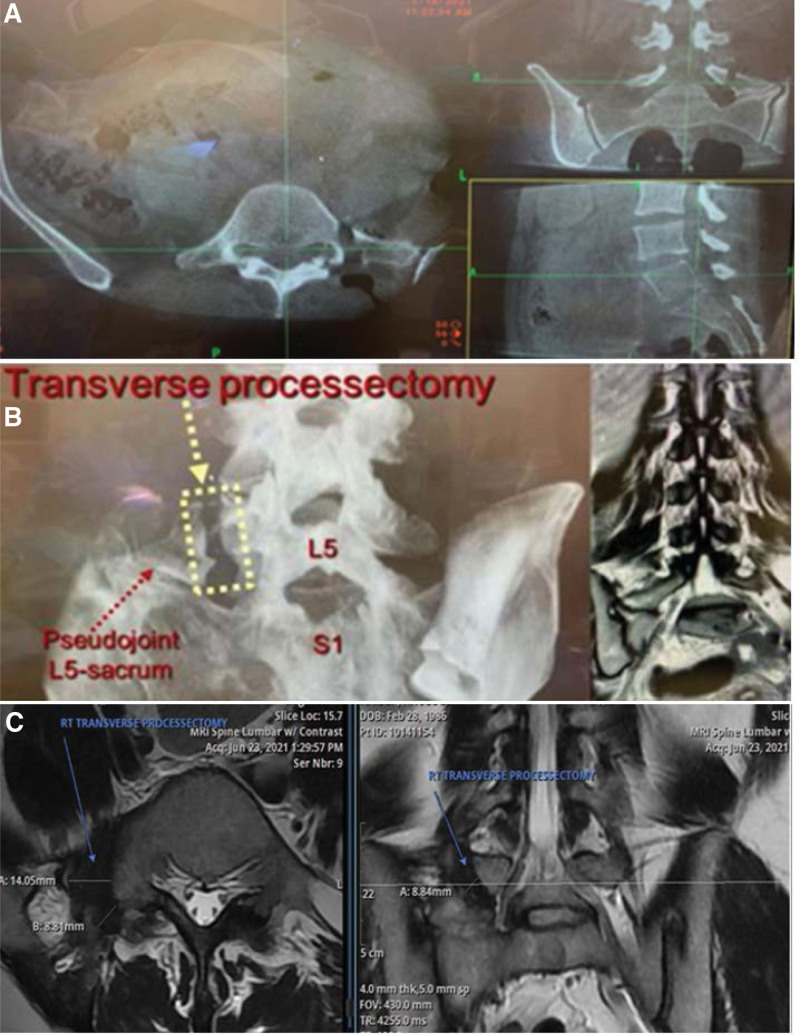
(A) Intraoperative 3-D image acquired by the *O-arm* (Medtronic^®^, USA) to confirm the complete disconnection of the enlarged transverse process (TP) from the pseudojoint sacrum region; (B) 3-D Image of the lumbar region showing the transverse processectomy (left) and preoperative lumbosacral MRI (right); (C) Postoperative lumbosacral MRI showing the transverse processectomy, axial (left) and coronal T2 image (right). TP = transverse process.

The surgeon should be cautious whenever approaching the inferior medial portion of the bone removal, as the nerve root of the inferior neuroforamen is passing very close to this part of the bone, sometimes being compressed by the malformed bone, and at this time it can be helpful to use an ultrasonic bone shaver or similar, and keep alert to any change on the intraoperative neuromonitoring (IONM) (Fig. 3). Completeness of the “ice hockey stick” shaped the bone gap can be confirmed using the real-time 3D intraoperative image to assure that there are no bone connections between the base of the enlarged TP, medially, and its lateral malformed portion (Fig. 4).

Washing inside the space with saline, bone wax is applied to the edges of the bone gap for haemostasis and to reduce the chance of delayed bone reunion. Vancomycin powder can be left intrawound (500 mg), closured by layers, and topical skin adhesive is applied on the skin, followed by the wound dressing. Patient can ambulate at the same day, whenever fully awake, and is usually discharged at the 2^nd^ postoperative day, taking oral analgesics, following general advices for wounds and postoperative spine care provided by the hospital team. The usual regular follow-up is on the 5^th^ postoperative day to change the wound dressing later, at 1, 3, 6, and 12 months postoperatively.

## 5. Results

A total of 16 patients with Bertolotti’s syndrome were included in this study, 8 patients were submitted to surgery for isolated BS, and 8 patients had an operation for BS and another associated lumbar disorder simultaneously. All patients complained of chronic paramedian (buttock region), low back pain, with or without radicular symptoms, not responding to the conservative treatment. According to the Castellvi radiographic classification,^[2]^ 16 cases (all cases) were classified as type II, 13 cases as II A, being 8 with the pseudoarticulation on the right side, 5 left on the side, and 3 cases as II B (bilateral pseudoarticulation) (Table 1 and 2).

**Table 1:** Data of patients with isolated Bertolotti’s syndrome submitted to tubular microsurgical transverse processectomy.

Patient	Age at time of surgery	Gender	Castellvi classification	Surgery performed (type and side)	Remarks: surgical complications
1.	40	F	II B; bilateral	Bilateral tubular microsurgical TP; using: IONM and *O-arm* to check the resection at the end	Very good result
2.	49	F	II B; bilateral	Bilateral tubular microsurgical TP	Very good result
3.	28	F	II A; RT	Tubular microsurgical RT TP	Very good result
4.	33	M	II A; RT	Tubular microsurgical RT TP; using: IONM, *O-arm* and neuronavigation (*L*3 spinous process) to check	RT LL after surgery
5.	34	F	II A; RT	Tubular microsurgical RT TP; using: IONM and *O-arm* to check the resection at the end	Wound (too small incision?) dehiscence requiring 1 debridement and dressing;
6.	32	F	II A; RT	Tubular microsurgical RT TP	Revision to remove remaining bone bridge during the same admission
7.	24	F	II A; LT	Tubular microsurgical LT TP; using: IONM and *O-arm* to check the resection at the end	Very good result
8.	38	M	II A; LT	Tubular microsurgical LT TP; using: IONM, *O-arm* and neuronavigation (reference frame taped on the skin) to check the resection at the end	Left foot pain for 2 wks, improved with analgesics, and had a good result

F = female, IONM = intraoperative neuromonitoring, LT = left, LSTV = lumbosacral transitional vertebrae, M = male, NN = neuronavigation, RT = right, TP = transverse process.

**Table 2:** Data of patients with Bertolotti’s syndrome and associated lumbar spinal disorder submitted to surgery.

Patient	Age at time of surgery	Gender	Castellvi classification	Surgery performed (type and side)	Remarks: surgical complications
1.	42	M	II B; bilateral	Bilateral mini-open transverse processectomy L5-LSTV, TLIF *L*4–5 LT	The vertical part of the RT TP reaches the sacrum, but no complication, partial pain improvement
2.	55	F	II A; RT	*L*2–3 decompression-microdiscectomy (RT) + *L*5 RT transverse processectomy	Recurrent RT leg-foot pain related to peroneal n syndrome
3.	51	M	II A; RT	MIS-mini-open TLIF *L*4–5 (RT) + RT transverse processectomy	Very good result
4.	46	F	II A; LT	Lumbar discectomy (October 2016 - Dubai); open *L*4–5-S1 TLIF + LT transverse processectomy	Persistent pain at Lumbar-LT side, unhappy
5.	34	F	II A; RT	Mini-open MIS TLIF *L*4–5 + RT transverse processectomy	Very good result
6.	36	F	II A; LT	*L*4–5 TLIF + LT transverse processectomy	Very good result
7.	38	F	II A; LT	*L*4–5 LT far lateral discectomy and tubular transverse processectomy	Very good result
8.	46	F	II A; RT	*L*5-LSTV revision of TLIF (operated in 2014 and 2015, UK) with pseudoarthrosis + RT transverse processectomy, using: IONM, *O-arm* and neuronavigation (reference frame fixed on the spinous process of *L*5) to check the implants and resection of the enlarged TP at the end	Good result

F = female, IONM = intraoperative neuromonitoring, LT = left, LSTV = lumbosacral transitional vertebrae, M = male, NN = neuronavigation, RT = right, TLIF = transforaminal lumbar interbody, TP = transverse process.

Among those 16 patients, 12 females and 4 males, with the average age of the patients at the date of surgery of 39.125 years (range 24–55 y.o.). The average follow-up after the surgery was 17 months (range: 3–36 months). Eight patients had isolated Bertolotti’s syndrome (which was the focus of this article), and the other 8 had another associated lumbar disorder requiring surgical intervention (Tables 1 and 2).

A total of 11 mini-open tubular microsurgical transverse processectomy (MO-TMTP) were performed for 8 patients (8/16) with isolated BS, which was the focus of this article, as the patients had only BS, avoiding confounding factors due to the associated lumbar disease and extra surgical procedures in the remaining 8 patients (8/16). The surgical technique used for this homogenous group of patients was a new modified MO-TMTP, performed bilaterally in 2 cases and reoperation for 1 patient because the bone resection was incomplete, identified at the postoperative CT scan. Intraoperative fluoroscopy was used in 6 cases (6/8) to locate the base of the enlarged TP; IONM was used in 5 patients (5/8), 3D intraoperative advanced spinal image (*O-arm*) with neuronavigation was used to localize the base of the pseudojoint to be removed and to check the final bone resection for the last 5 cases (5/8).

Among those 8 patients, 4 of them had only paramedian (buttock region) lower back pain; 4 patients had radicular pain associated with paramedian lower back pain; 3 had type II A with ipsilateral radicular pain; and 1 had type II B with bilateral radicular pain (Table 1, 3).

**Table 3:** VAS for LBP (paramedian); RT and LT LL; before and after tubular microsurgical transverse processectomy for isolated Bertolotti’s syndrome.

Patient	VAS lumbar (central-paramedian) before surgery	VAS lumbar (central-paramedian) after surgery	VAS RT LL before surgery	VAS RT LL after surgery	VAS LT LL before surgery	VAS LT LL after surgery
1.	7	3	0	0	0	0
2.	7	1	4	0	4	0
3.	6	0	0	0	2	2
4.	7	0	0	0	0	7
5.	5	0	6	0	0	0
6.	7	3	0	0	0	0
7.	6	3	0	0	0	0
8.	8	2	0	0	6	1

F = female, IONM = intraoperative neuromonitoring, LBP = low back pain, LT = left, LSTV = lumbosacral transitional vertebrae, M = male, NN = neuronavigation, RT = right, TLIF = transforaminal lumbar interbody, TP = transverse process, VAS = visual analogue scale.

The average paramedian lower back pain before surgery on the visual analogue scale (VAS) for pain in the 8 patients was 6.6 (range: 5–8) and reduced to 1.5 (range: 0–3) at the latest follow-up after surgery, while the average pain score of the radicular pain before surgery was (range: 0–6) and reduced to 0.6 (range: 0–7) after the surgery 1.3.

Among those 16 patients, 2 intraoperative complications happened (2/16). One patient who had isolated BS had incomplete bone resection, leaving a small bridge of bone identified by a routine postoperative CT scan, for whom it was performed another surgery 3 days later, during the same admission, to complete the bone removal, but the 3D intraoperative spinal image was not used in this case. In another case, who had bilateral transverse processectomy associated with lumbar fusion, it was noticed by a routine postoperative CT scan that the inferior part of the bone resection was too inferior, reaching the superior portion of the sacrum, but asymptomatic.

Two postoperative adverse events were noticed in this series of 16 patients. In one case, where it was used a smaller surgical approach for the transverse processectomy, only + 2.5 cm, the patient experienced wound dehiscence, which was treated with surgical debridement and closure. Another patient who had transverse processectomy for chronic paramedian lower back pain, using a 3D intraoperative spinal image to check the bone removal, with no relevant nerve abnormalities noticed during the surgery by IONM, developed significant radicular pain after surgery. The pain was described as moderate to severe, sharp, burning, and constant. There was diminished sensation in the *L*5 dermatome and evidence of hyperesthesia, but motor strength was preserved. Postoperative lumbosacral X-ray, CT, and MRI scans were performed after surgery, which did not demonstrate any significant hematoma, fluid collection, bone fragments, nor compression on the nerve roots, suggesting that it was related to an inadvertent intraoperative nerve manipulation during the transverse processectomy. The patient was treated conservatively with analgesics, physiotherapy, and lumbar spinal injection and experienced gradual relieve of his radicular pain only after 4 months of postoperative (Table 3).

## 6. Discussion and conclusion

In our patient series type II was the most common type associated with LBP, and, the level above the transitional vertebrae was the most common level that has disc disease, same result concluded by Castellvi et al and in other study.,^[2],[21]^.

Limited understanding of the pathophysiology and exact etiology of Bertolotti’s syndrome has led to a lack of uniformity in the diagnosis and treatment. Images alone will not help to diagnose the pain generator, recommending the use of a diagnostic block tests of the pseudo articulation or the nerve for additional diagnostic information. Almeida et al proposed a diagnostic therapeutic algorithm to help in decision making in patients with back pain associated with LSTV,^[22]^ which was used to guide the management of the patients of this study. Some attributed this pain to changes in load transmission through the spine and thus changes in bone, ligament functions and morphology,^[23]^ nerve compression by disc herniation or entrapment of the lumbar nerve under the mega transverse process or arthritic changes from pseudoarticulations.^[24]^ Bertolotti^[2]^ suggested LBP due to arthritic changes occurring at the site of pseudoarthrosis. Connolly et al^[25]^ demonstrated that 80% of young patients with low back pain and LSTV had high uptake on bone scintigraphy at the transverse process sacral articulation. In our study, it seems that the main cause of pain was the pseudo-articulating joint as all patients experienced short-term pain relief after the injection test using local anesthetic plus steroids, and a good results for most patients after the transverse processectomy which can be attributed to removal of the load from the pseudoarticulated joint, and the same for relief of the radiculopathy may be it is due to decompression of the nerve by transverse processectomy.

Treatment of Bertolotti’s syndrome range from conservative treatment including trial of pain management and physical therapy until surgery for refractory cases. Temporary pain relief can be achieved by local injection of local anesthesia with steroid^[26]^ and by radiofrequency ablation by creating a strip lesion at the pseudoarthritic joint.^[27]^ There are different surgical options for the surgical treatment of BS, including lumbar fusion or resection of the pseudoarthritic joint. Surgical resection of part of the enlarged TP, to disconnect the lumbar segment from the malformed pseudojoint, and unload the pseudoarticulation seems to be a good option in selected patients, with potential good outcomes,^[28–31]^. Lumbar spinal fusion also shows a good result, according to the case and associated disc disorder^[32]^. The outcome of posterolateral fusion was reported by Santavirta et al, who showed no significant differences compared with resection of the pseudoarticulation.^[33]^.

Takata et al, describe a technique using an endoscope and burr for removal of the pseudojoint with successful pain relief.^[34]^ The article of Li Y et al,^[29]^ described the minimally invasive tubular resection of the anomalous TP in patients with BS, however, they suggested the bone removal to disconnect the enlarged TP in a horizontal fashion. The modified technique suggested by the authors of this study proposes to start removing the base of the TP vertically, instead, around 1 cm lateral to the pedicle, creating a bone gap of at least 5 to 10 mm, lateral to the base of the TP. After this partial bone resection lateral to the pedicle, the bone resection is re-directed inferior-medially, below the pedicle, on “ice hockey stick” shape form, around 1 cm from the base of the enlarged TP. This technique offers the advantage of cutting the bone in a more vertical manner, close to the pedicle, avoiding extending the bone removal laterally, which according to the size and shape of the malformed TP, may require an unpredictable lateral extension of the bone resection (Fig. 3).

While our suggested technique shown good improvement in pain score, still there is limitations of our study, the retrospective fashion of our study, the small sample number of the patients who had isolated BS and underwent only modified MO-TMTP, and relatively short period of follow up. Larger prospective studies and longer duration follow up are needed to analysis the results of this surgical technique.

## 7. Conclusion

A new modified MO-TMTP, guided by 3D intraoperative image and IONM proposed in this paper seems to be a safe and effective procedure for selected patients with BS, refractory to conservative treatment, who experienced a temporary pain relief after injection of pseudo articulation with steroids and local anesthetic. Larger prospective studies are needed to analysis the results of this surgical technique.

## Author contributions

**Conceptualization:** Hatem Afana, Muhammad Raffat.

**Data curation:** Nicandro Figueiredo.

**Investigation:** Hatem Afana.

**Methodology:** Hatem Afana.

**Project administration:** Hatem Afana.

**Supervision:** Nicandro Figueiredo.

**Validation:** Hatem Afana, Nicandro Figueiredo.

**Writing – original draft:** Hatem Afana, Muhammad Raffat.

**Writing – review & editing:** Hatem Afana, Muhammad Raffat, Nicandro Figueiredo.
